# Association between Global Life Satisfaction and Self-Rated Oral Health Conditions among Adolescents in Lithuania

**DOI:** 10.3390/ijerph14111338

**Published:** 2017-11-03

**Authors:** Aistė Kavaliauskienė, Antanas Šidlauskas, Apolinaras Zaborskis

**Affiliations:** 1 Clinic of Orthodontics, Faculty of Odontology, Medical Academy, Lithuanian University of Health Sciences, 50106 Kaunas, Lithuania; antanas.sidlauskas@lsmuni.lt; 2 Department of Preventive Medicine & Health Research Institute, Faculty of Public Health, Medical Academy, Lithuanian University of Health Sciences, 47181 Kaunas, Lithuania; apolinaras.zaborskis@lsmuni.lt

**Keywords:** quality of life, life satisfaction, oral health, oral health-related quality of life, adolescents, child perceptions questionnaire

## Abstract

*Background*: This study aims to explore the extent to which the perceived oral conditions predict adolescent global life satisfaction (GLS); *Methods*: The sample in a cross-sectional survey consisted of 1510 Lithuanian adolescents (41.7% boys) aged 11–18. The survey was conducted by means of self-report questionnaires that were administrated in school classrooms ensuring confidentiality and anonymity of the participants. The schoolchildren rated their GLS and answered the questions about perceptions of their oral health. The relationship between GLS and oral health variables was estimated using unadjusted and adjusted binary logistic regression and nonparametric correlation analyses; *Results*: The research showed that the majority of adolescents rated their GLS highly; however, girls, older adolescents and adolescents from less affluent families were less likely to report high scores. GLS was significantly associated with subjective overall oral health assessment. The odds of reporting low GLS were 50% higher for adolescents with good oral health (OR = 1.51; *p* < 0.001; 95% CI = 1.18–1.93), and two and half time as higher for adolescents with perceived fair/poor oral health (OR = 2.78; *p* < 0.001; 95% CI = 1.72–4.50) compared to adolescents with subjectively excellent/very good oral health. Nonparametric correlations indicated lower GLS to be significantly associated with higher scores of Child Perceptions Questionnaire (𝜌 = −0.17/−0.30; *p* < 0.01); *Conclusions*: Adolescents with oral health impairments more likely to report lower GLS, regardless of their gender and age.

## 1. Introduction

Life satisfaction is considered a key aspect and a positive psychological dimension of quality of life (QoL) [1]. During adolescence, it is strongly influenced by life experiences and relations, particularly in the family, in the school environment and with peers [2,3,4,5,6]. On the other hand, better life satisfaction may act as a buffer against the negative psychological dimensions such as anxiety, depressive and stress symptoms [7,8,9]. The assessment and improvement of psychosocial well-being of young people is a great concern of health care including dentistry [8]. 

In general, most adolescents report positive life satisfaction regardless of a high cross-cultural variation in their response prevalence [10]. The 2016 Health Behaviour in School-aged Children (HBSC) study covering 41 countries has revealed that prevalence of positive life satisfaction significantly declined between ages 11 and 15, with boys reporting a significantly higher prevalence, and family affluence was significantly positively associated with high life satisfaction in nearly all countries [10]. 

There is evidence of a close relationship between life satisfaction of adolescents and their subjective health and health-related quality of life [11,12,13]. In particular, a study of Zullig et al. [12] found that adolescents, aged 13–18 years, who declared to have more days of poor physical or mental health in the past 30 days, showed lower life satisfaction, independently of gender. Notwithstanding this, mental health appears to make a greater contribution to life satisfaction and QoL [14]. 

Emerging consensus in literature has identified oral health-related quality of life (OHRQoL) as a multidimensional construct containing physical, social and psychological domains [15,16]. Although oral disorders can impact many aspects of these domains, the OHRQoL and its categories can be measured through questionnaires with different methodologies [17]. In 2002, Jokovic et al. [18] developed the Child Perceptions Questionnaire (CPQ), which is one of the first and the most widely used inventory designed to assess the impact of oral conditions on QoL in children and adolescents. In particular, the term of OHRQoL has been seen increasingly used in the orthodontics literature [19,20,21]. It has been recognized that individuals with malocclusions often feel self-conscious in social situations and may have facial and dental appearance-related self-concept issues. Therefore, it is reasonable to expect that orthodontic anomalies should result in reduced self-esteem and overall life satisfaction. Much of the orthodontic treatment is justified on the basis of improving psychosocial well-being and OHRQoL [21]. However, several studies have argued that malocclusion and orthodontic treatment do not appear to affect general or OHRQoL to a measurable degree, despite subjective and objective evidence for improved appearance, oral function, health, individual life satisfaction and social well-being [22,23]. 

Thus, the reality of association between overall QoL of young people and their oral health status is not clear cut and the research in this area still leaves many questions unanswered. The evidence is frequently conflicting due to differences in research design, methods of assessment of psychological health and the age of populations studied. By the age of 11 or 12, children view health as a multidimensional concept with the following constructs: being functional, adhering to good lifestyle behaviours, a general sense of well-being and relationships with others [18,24,25]. This indicates that starting at this age a child can be asked to assess both health status and well-being by means of one question. Clearly, they need to be phrased to accommodate child’s age-dependent understanding of health and well-being, e.g., using the “Cantril ladder” [26]. Jokovic et al. proposed a single-question approach for self-rating of the overall oral health and the extent to which the oral/orofacial condition affected his/her oral health related well-being [18,24]. In the HBSC study a measure of global life satisfaction (GLS) was also designed on a single-question [10]. 

It is not clear what lies behind the subjective evaluation of GLS. Overall, do young people take in mind their perceptions of oral health while rating their GLS? If yes, then to what extent? To the best of our knowledge, to date there are few studies [27,28,29] assessing the association between oral health conditions and OHRQoL in representative samples of children and adolescents, and there are no studies assessing their association with self-reported global happiness or life satisfaction. Consequently, we focussed on adolescents’ perceptions of oral/orofacial conditions and their perceived QoL in the current study. We examined the relationship between GLS in adolescents and various oral health variables including self-reported rating of malocclusion and effect of undergoing orthodontic treatment. The aim of this study was to explore the extent to which the perceived oral conditions predict adolescent GLS. 

## 2. Methods 

### 2.1. Subjects and Study Procedures 

The present study was a part of a research project aimed to examine oral health and OHRQoL among children and adolescents in Lithuania. The subjects and study procedures of the present study were the same as in the main project. The study followed a cross-sectional design and targeted adolescents aged 11 to 18 years. A sample size of 1614 persons was calculated to be required for assessment of the prevalence of orthodontic anomalies as the main factor of this research project, hypothesizing its prevalence to be 30% with 95% confidence interval from 27.5% to 32.5%, as well as accounting for anticipated non-response of 20%. The sample was made up of students from government schools of general education in Lithuania. A list of schools was obtained from the education management information system of the Lithuanian Centre of Information Technologies in Education. 

A two stage random cluster (school, class) sampling technique was applied in the study. In the first stage, primary sampling units were schools which were selected randomly from the list of schools in Lithuania. The 27 selected schools represented all regions of the country and warranted the sufficient number of students to be recruited into the study sample. This was estimated by gathering information from schools about the number of students and classes from the 6th to 11th grades. A list of all the classes by grade was prepared separately for each selected school. In the second stage of sampling, the students’ classes were selected with a probability proportionate to the number of students in school. In comparison with other grades, the classes of the 9th and 11th grades had higher priority to be selected as students of this age were in focus with regard to other objectives of the research project [30]. The selection of classes continued until the required sample of students was drew. 

The authorities of selected schools were contacted by researchers and informed about all aspects of the study. In each chosen class, students’ parents were asked to give a permission for their children to participate in the study. Of the 1896 initially invited parents, written informed consents approving each child’s participation in the study were obtained from 1652 (87%) parents. Then, 1586 students (96% of those who had parents’ permission) completed anonymous questionnaires. The questionnaires were administrated in school classrooms by ensuring respondents’ confidentiality and anonymity. They were carried out according to the standard instructions by teachers. All the eligible and willing students in that classroom were given the questionnaires to prevent discrimination. A total of 1510 students responded to all questions of interest in the present analysis.

A pilot test was carried out on a sample (N = 48) of children in one school. It confirmed the feasibility of the methodology with only minor modification of wording of the questionnaire, mainly paying attention of respondents to the relation of symptoms to their oral health. The main survey was conducted in the 2013/2014 school-year. 

### 2.2. Instrument and Variables

The originally created self-reported questionnaire consisted of items assessing different aspects of general and oral health, health behaviour and OHRQoL. Several items and scales were also adopted from the other studies (see below).

#### 2.2.1. Global Life Satisfaction 

Child’s GLS, or well-being, was rated using the measurement technique from the HBSC study [10]. Children were asked to take a look at the drawn ladder, with steps numbered from zero (“0”) at the bottom to ten (“10”) at the top, with the instruction to suppose the top of the ladder represented the best possible life, and the bottom of the ladder represented the worst possible life. Then they were asked to indicate the step of the ladder at which they would place their lives at present. Thus, the response was scored from 0 to 10. In analyses, the item was also dichotomised into ‘low GLS’ (0–7 scores) and ‘high GLS’ (8–10 scores). 

#### 2.2.2. Family Affluence

Family affluence was measured by the Family Affluence Scale (FAS), which has been specially developed for the HBSC study as a measure of social position [10]. The scale is simple and easy to answer even for children. The present FAS includes four questions, which cover cars and home computer ownership, own bedroom occupancy and travelling on holidays. A composite FAS score was calculated for each respondent based on his responses to these four items and then a three-point ordinal variable was composed for the present analysis in which: score = 0–3 indicated low affluence; score = 4–5 indicated middle affluence, and score = 6–7 indicated high affluence. 

#### 2.2.3. Dental Fear 

We asked the respondents the following question: “How much are you afraid of dental treatment?”. Respondents could choose one of the five answers: 1 = not at all, 2 = a little, 3 = somewhat, 4 = much, 5 = very much. In analyses, the outcome was dichotomized as ‘little dental fear’ (for answers 1 and 2) and ‘great dental fear’ (for remaining answers).

#### 2.2.4. Rating of Oral Health and Well-Being 

The respondents were asked to rate their oral health and the extent to which it affected their well-being. For each of these dimensions five sub-items were worded in the following way: “How would you describe health status of the following oral parts: —teeth; —lips; —gum; —oral mucosa; —jaws or joints?” and “Over the last three months, how much your overall life was affected by the conditions of the following oral parts: —teeth; —lips; —gum; —oral mucosa; —jaws or joints?” The responses were scored in the following way: with regard to an oral health rating: 0 = excellent, 1 = very good, 2 = good, 3 = fair and 4 = poor; with regard to well-being: 0 = not at all, 1 = very little, 2 = somewhat, 3 = a lot and 4 = very much. The final score computed the maximal score on all the sub-items of each dimension. In regard to the distribution of ratings, the following two items were constructed: ‘overall oral health assessment’ with categories ‘excellent/very good’, ‘good’, ‘fair/poor’, and ‘oral conditions affected well-being’ with categories ‘not at all’, ‘very little/somewhat’, ‘a lot/very much’. 

#### 2.2.5. Child Perception Questionnaire 

The originally proposed ‘Child perception questionnaire (CPQ)’, is a 37-item scale consisting of four health domains (subscales), namely oral symptoms (OS, 6 items), functional limitations (FL, 9 items), emotional well-being (EWB, 9 items), and social well-being (SWB, 13 items) [18]. The items are scored on a 5-point Likert scale ranging from 0 (“never”) to 4 (“every day or almost every day”). In the analysis, the scores for each item were added together to obtain a sum scores of each sub-scale as well as the total CPQ scale. Then, the sum scores were standardized to a percentage score scale of 0–100% by dividing the sum score by the maximum score and multiplying by 100. Note that higher sum/percentage scores refer to worse OHRQoL.

#### 2.2.6. Self-Reported Rating of Caries Experience and Malocclusion 

Respondents were asked to answer: (1) whether they had dental caries (tooth decay) or cavities to be treated, and (2) whether they had ever noticed that their teeth were irregularly grew/situated or they had malocclusion. The answer categories were: 1-yes, I noticed myself only; 2-yes, this was confirmed by dentist; 3-no, I don’t have such disorders. 

#### 2.2.7. Completed Orthodontic Treatment and Its Perceived Effect 

Those respondents who reported possible or confirmed by a dentist malocclusion problems, were asked additionally whether they have had an orthodontic treatment. The question was asked separately with regard to dental plate and fixed orthodontic appliance (braces) therapy. If the treatment was completed, then patients were asked to evaluate its effect by rating its helpfulness: 1 = it was greatly helpful; 2 = it was moderate helpful; 3 = it is difficult to say if it was helpful; 4 = it was not helpful; 5 = it was not at all helpful; and 6 = I don’t know results as treatment just started. In analysis, treatment effect was dichotomized into ‘achieved positive effect’ (1 and 2 answer options) and ‘not achieved positive effect’ (3, 4 and 5 answer options). Respondents of 6 answer option without completed orthodontic treatment were not included into treatment effect analysis. 

### 2.3. Statistical Analysis

Missing data of the CPQ items was replaced with the personal mean if a health domain had not more than half blank items, otherwise the record was excluded from analysis. The distributions of the sum scores of subscales and total scale as well as distribution of the GLS scores were examined and found not to be normally distributed, thus, binary associations between variables were evaluated with non-parametric Spearman correlation coefficient. Chi-squared test was applied to assess the differences in the prevalence of high and low GLS between the groups of respondents. Binary logistic regression analysis was used to produce unadjusted and adjusted odds ratios (OR) with 95% confidence intervals (95% CI), which indicated the likelihood of having low GLS for adolescents with certain characteristics relative to the reference group. Adjusted odds procedure (multivariate logistic regression) controlled for the effect of other characteristics, including sociodemographic indicators: gender, age and family wealth. All reported *p* values were from two-sided statistical tests and *p* values ≤ 0.05 were considered statistically significant. The whole analysis was performed using the Complex Samples module of the SPSS statistical package (version 20; IBM SPSS Inc., Chicago, IL, USA, 2011) which adjust for the complex cluster-stratified sampling method (schools, classes) and weighted data [31].

### 2.4. Ethical Statement

The study was conformed to the principles outlined in the Declaration of Helsinki. Ethical approval for the study was granted by the Kaunas Regional Biomedical Research Ethics Committee (reference number BE-2-27). In line with local practice for general school surveys, the study was agreed with national and local educational institutions. Additionally, written informed consent for child’s participation in the study was sought from both parents.

## 3. Results

### 3.1. Sample Characteristics

A total of 1510 students, 629 (41.7%) boys and 881 (58.3%) girls, responded to all questions of interest in the present analysis. Mean age was 15.86 years (SD = 1.57). The respondents were distributed in three age groups (11–14, 15–16, and 17–18 years) which sample sizes were sufficient to obtain an appropriate statistical significance of differences in studied characteristics by age groups. Table 1 shows descriptive statistics of other sample characteristics.

### 3.2. Global Life Satisfaction, Socio-Demographic and Oral Health-Related Variables 

The majority of adolescents rated their GLS so highly that the total distribution of scores was found to be skewed and not to be normally distributed (skewness = −1.03). The scores of the GLS ranged between 0 and 10 and the mean score was 7.72 (SD = 1.49), median was 8 (IQR = 2). Of 1510 adolescents, 62.8% rated their GLS by 8 scores or more (high GLS), while the remaining 37.2% rated their GLS at more than 7 scores (low GLS). These proportions were significantly related with studied determinants (Table 2). 

The rate of low GLS was higher among girls than among boys (40.6% vs. 32.3%; *p* < 0.001). There were also age-related and family wealth related differences in GLS scores distribution showing low GLS more common among older adolescents and adolescents from less affluent families. Adolescents who reported a high fear of dental treatment were more likely to indicate low GLS too.

Unadjusted (univariate) analysis indicated that the distribution of GLS scores was found to be significantly associated with several variables of self-rated oral health. The assessment of overall oral health as fair/poor and recognition that oral conditions affected personal well-being a lot/very much were related with the highest rate of low GLS. A caries experience and malocclusion seemed also to be associated with lowered GLS scores, however, significantly higher rate of low GLS was detected only in adolescents who subjectively felt a problem in their oral health but not confirmed yet by dentist. After the adjustment for possible confounders and other variables, the odds of reporting low GLS were 50% higher for students with good oral health (OR = 1.51; *p* < 0.001; 95% CI = 1.18–1.93), and two and half time as higher for students with perceived fair/poor oral health (OR = 2.78; *p* < 0.001; 95% CI = 1.72–4.50), compared to students with subjectively excellent/very good oral health. The variables such as oral conditions affected well-being, self-reported rating of caries experience and self-reported rating of malocclusion were not as significant as they were in unadjusted analysis.

### 3.3. Global Life Satisfaction and CPQ Scores 

Nonparametric correlation analysis identified a significant association between lowered GLS scores and increased sum score of the total CPQ and its four domains (Table 3). In all adolescents, regardless of their age, the strongest correlation was with the emotional well-being domain (𝜌 = −0.27; *p* < 0.01) but among boys this domain (𝜌 = −0.20; *p* < 0.01) was overtaken by the domain of oral symptoms (𝜌 = −0.27; *p* < 0.01).

### 3.4. Global Life Satisfaction and Effect of Completed Orthodontic Treatment 

Table 4 shows prevalence of low GLS in a group of adolescents with malocclusion (dentist confirmed) who had completed orthodontic treatment (N = 445), by self-reported effect of treatment. The findings show that those adolescents who felt a great/moderate improvement of their malocclusion (achieved a positive effect of orthodontic treatment) reported low GLS almost in the same rate as healthy adolescents (37.3% vs. 34.7%). Those adolescents who had completed treatment but did not feel any improvement of their malocclusion (not achieved a positive effect of orthodontic treatment) were more likely to report lowered GLS compared to the adolescents from the first group, although statistical significance of the difference in rates of low GLS between groups was not still sufficient due to small sample size (44.4% vs. 37.3%; *p* = 0.172). 

## 4. Discussion

The present study was focused on testing of a very clear hypothesis about the moderating role of oral health conditions and their perceptions on the GLS in adolescents. The results confirmed this hypothesis suggesting that adolescents with impairment of oral health are more likely to report lower GLS, regardless of their gender and age. 

In general, the majority of adolescents in the present study reported high GLS, which is in accordance with the results obtained in other studies [6,10,12,32,33]. Consistent gradients were found for life satisfaction in respect to gender, age and family affluence, confirming findings from the HBSC research [10]. Our study revealed also a significant relation between GLS and dental fear that is in line with results of the studies by Merdad and El-Housseiny [34] and Schuch et al. [35] who demonstrated a significant impact of dental fear on OHRQoL among schoolchildren. 

The study was complicated by the fact that adolescents have very different concepts of QoL and life satisfaction when compared with adults [36,37]. It is widely acknowledged that a child’s own concepts of QoL change with age; hence, it is often difficult to separate changes due to normal development from those due to disease treatment. Another challenge is cognitive development in understanding of questions and self-rating of health and life satisfaction concerns to a specific scale. Additionally, the majority of measures developed in the field of child dentistry has tended to concentrate on OHRQoL rather on general QoL, regardless of differences in research design, populations studied and methods of assessment of assessment of child well-being [1,17,20].

Despite the conflicting aspects mentioned above, there is an increasing recognition that oral health has a significant impact on physical, social and psychological well-being [15,16,17]. At the same time the mechanisms behind association studied are complex and likely to result from several interwoven processes. Among young people, there is more evidence in relations between OHRQoL and oral disorders, although most of published papers in this field of research describe results from cross-sectional surveys [38]. Kiyak’s 2008 review of research on the impact of malocclusion and its treatment on OHRQoL examined the evidence for and against this association [23]. Some authors strongly argue that youths with high clinical need of orthodontic treatment report significantly poorer OHRQoL than the patients with less critical treatment need, but mainly on the emotional and the social well-being domains [39,40]. Furthermore, definite malocclusion has a negative correlation with subjective happiness of young adolescents [41]. Another hypothesis suggests that aesthetic appearance of the mouth and smile may also play an important role in adolescent social interactions and psychological well-being [27,29,42]. An unpleasant facial appearance may stigmatize a person, hinder school performance, encourage negative stereotypes, and cause a negative effect on self-esteem, happiness and life satisfaction [41,43]. Finally, young patient benefits psychologically rather than clinically from the orthodontic treatment with improved facial and dental appearance [44].

In the present study, we used the CPQ that was proposed by Jokovic et al. [18] as an inventory to measure OHRQoL among children aged 11–14 years. Although this inventory we used for respondents within a broader age range, the study results revealed a significant correlation between GLS and all domains of the CPQ among adolescents of all age groups. These findings highlight the positive association between GLS and OHRQoL among adolescents. These results represent novelty of our study as to our knowledge the association between QoL and oral health status in adolescents had never been taken into consideration to date. 

The current study gives new insides into the QoL determinants in young people indicating that adolescent perceptions of oral health disorders have a significant impact on his GLS. This finding has important implications because it can enhance understanding of how oral conditions affect the life of adolescents. Dental caries and malocclusion have relatively high prevalence among adolescents of Lithuania [30,45,46]. It is thus important to know how this impacts on adolescent day-to-day lives and whether changes in dental care may affect this. The study results show also several specific insights. One of such insights concerns adolescents who subjectively felt a problem in their oral health but not confirmed yet by the dentist. These adolescents reported significantly lower GLS compared with their counterparts, including that those oral health problems have been already confirmed by dentist. This finding behaves the general dentist and orthodontist to listen carefully to each adolescent patient as early as possible understanding of his or her oral health problems and their impact on the quality of life, including oral function appearance, emotional well-being and social acceptance, as well as to undergone effective treatment in early childhood or adolescence. On the other hand, health promotion in young people should also initiate a continuous oral health checking and lowering dental fear at clinic. 

### Study Strengths and Limitations

The major strength of the present study lies in the use of standardized variables and scales validated in the well-known studies such as the HBSC study (GLS and FAS measures) [10] and OHRQoL studies (CPQ measures) [18,24]. Another important aspect is that this study, unlike the usual tendency of being carried out on convenience samples, was conducted on a sample representative of an entire population, which implies a sample of adolescents who represent the whole society at all its socio-demographic levels. This aspect provides good evidence for generalization of study results back to the population. 

Some limitations of the study need to be considered as well. As the present study was a cross-sectional survey, there was no possibility of evidencing a causal link in the association found, such as between GLS and oral health conditions. Longitudinal studies are necessary for better understanding the relationship between the variables studied. The study is also limited in a single-question measure of GLS. This has the disadvantage over multi-item measures as the single answer does not provide information about different dimensions of life satisfaction that can be useful for clinical decision-making purposes, i.e., treatment planning [24]. In this article we used subjectively rated health status; the future our studies, however, will provide objectively measured information on dental caries and orthodontic treatment need. Other limitations are related with sampling approaches. The adolescents from a general population (from schools only but not from dental clinics) were recruited into the survey sample. This resulted in a low percentage of respondents with impairment of oral health, including individuals with completed orthodontic treatment and, consequently, insufficient sample size to test hypotheses related with treatment effects. We also were not able to evaluate a difference between respondents and non-respondents who or their parents declined participation in the survey. It is possible that non-respondents would have different social characteristics and profiles in relation to the outcome and its determinants, and so the study estimates may be biased [47]. However, our study succeeded in relatively low (just 13%) of non-respondents owing to negative parent consent. 

## 5. Conclusions

Results of our study confirmed an association between GLS and self-rated oral health conditions among adolescents in Lithuania, i.e., that adolescents with impairment of oral health are more likely to report lower global life satisfaction, regardless of their gender and age. This finding highlights that the effect of oral health perceptions on life satisfaction in adolescents should be taken into consideration in studies of young people quality of life. 

## Figures and Tables

**Table 1:** Descriptive characteristics of a sample of adolescents in Lithuania (N = 1510) ^a^_._

Characteristic	No. (%) of Respondents	
Gender:		
boys	629	(41.7)
girls	881	(58.3)
Age group (years):		
11–14	319	(21.2)
15–16	686	(45.4)
17–18	505	(33.4)
Family affluence:		
high	749	(50.8)
medium	547	(37.1)
low	178	(12.1)
missing	36	
Fear of dental treatment:		
little	910	(60.3)
great	599	(39.7)
missing	1	
Overall oral health assessment:		
excellent/very good	844	(55.9)
good	560	(37.1)
fair/poor	106	(7.0)
Oral conditions affected well-being:		
not at all	532	(35.2)
very little/somewhat	838	(55.5)
a lot/very much	140	(9.3)
Self-reported rating of caries experience:		
healthy	986	(65.5)
not healthy (dentist confirmed)	219	(14.6)
not healthy (subjective feeling)	300	(19.9)
missing	5	
Self-reported rating of malocclusion:		
healthy	562	(37.5)
not healthy (dentist confirmed)	585	(39.1)
not healthy (subjective feeling)	350	(23.5)
missing	13	
Global life satisfaction:		
high	949	(62.8)
low	561	(37.2)
mean (SD)	7.72	(1.49)
median (IQR)	8	(2)
CPQ: mean (SD):		
oral symptoms	22.05	(16.77)
functional limitations	6.76	(10.84)
emotional well-being	11.66	(17.88)
social well-being	2.97	(7.55)
total CPQ	9.10	(9.24)

^a^ Abbreviations: IQR—interquartile range; SD—standard deviation; CPQ—child perception questionnaire.

**Table 2:** Prevalence of low global life satisfaction and its odds in a sample of adolescents in Lithuania ^a^_._

Characteristics	No. (%) of Adolescents		OR (95% CI)	
	With Low GLS (N = 561)	Total (N = 1510)	Unadjusted	Adjusted
Gender:				
boys	203 (32.3)	629	1	1
girls	358 (40.6)	881	1.44 (1.16–1.78) ***	1.40 (1.11–1.76) **
*p* ^b^	0.001			
Age group (years):				
13–14	102 (32.0)	319	1	1
15–16	249 (36.3)	686	1.21 (0.91–1.61)	1.24 (0.96–1.74)
17–18	210 (41.6)	505	1.51 (1.13–2.03) **	1.55 (1.14–2.12) **
*p* ^b^	0.017			
Family affluence:				
high	242 (32.3)	749	1	1
medium	210 (38.4)	547	1.31 (1.04–1.64) *	1.26 (1.00–1.60)
low	99 (55.6)	178	1.63 (1.88–3.66) ***	2.34 (1.65–3.31) ***
*p* ^b^	<0.001			
Fear of dental treatment:				
little	307 (33.7)	910	1	1
great	254 (42.4)	599	1.45 (1.17–1.79) ***	1.29 (1.03–1.62) *
*p* ^b^	0.001			
Overall oral health assessment:				
excellent/very good	261 (30.9)	844	1	1
good	240 (42.9)	560	1.68 (1.34–2.09) ***	1.51 (1.18–1.93) ***
fair/poor	60 (56.6)	106	1.91 (1.93–4.39) ***	2.78 (1.72–4.50) ***
*p* ^b^	<0.001			
Oral conditions affected well–being:				
not at all	159 (29.9)	532	1	1
very little/somewhat	337 (40.2)	838	1.58 (1.25–1.99) ***	1.28 (1.00–1.65)
a lot/very much	65 (46.4)	140	2.03 (1.39–2.97) ***	1.07 (0.68–1.67)
*p* ^b^	<0.001			
Self–reported rating of caries experience:				
healthy	349 (35.4)	986	1	1
not healthy (dentist confirmed)	78 (35.6)	219	1.10 (0.74–1.37)	0.84 (0.60–1.16)
not healthy (subjective feeling)	131 (43.7)	300	1.42 (1.09–1.84) **	1.02 (0.77–1.37)
*p* ^b^	0.031			
Self–reported rating of malocclusion:				
healthy	195 (34.7)	562	1	1
not healthy (dentist confirmed)	217 (37.1)	585	1.11 (0.87–1.41)	0.96 (0.74–1.24)
not healthy (subjective feeling)	144 (41.1)	350	1.32 (1.01–1.73) *	1.13 (0.84–1.51)
*p* ^b^	0.147			

^a^ Abbreviations: GLS—global life satisfaction; OR—odds ratio; CI—confidence interval. ^b^ Chi square test. * *p* < 0.05; ** *p* < 0.01; *** *p* < 0.001.

**Table 3:** Spearman correlation coefficients ^a^ between the ratings of global life satisfaction and the CPQ domain scores, by gender and age groups.

Domain/Scale	All Adolescents	Gender		Age Group (Years)		
		Boys	Girls	13–14	15–16	17–18
Oral symptoms	−0.23	−0.27	−0.19	−0.27	−0.23	−0.20
Functional limitations	−0.17	−0.18	−0.14	−0.16	−0.17	−0.19
Emotional well-being	−0.27	−0.20	−0.28	−0.31	−0.25	−0.29
Social well-being	−0.19	−0.15	−0.21	−0.19	−0.18	−0.22
Total CPQ	−0.30	−0.29	−0.29	−0.33	−0.29	−0.31

^a^ All estimations significant at *p* < 0.01.

**Table 4:** Prevalence of low global life satisfaction in a group of adolescents with malocclusion (dentist confirmed) who had completed orthodontic treatment, by self-reported effect of treatment ^a^.

Self-Rated Effect of Orthodontic Treatment	No. (%) of Adolescents with Low GLS (N = 175)	Total (N = 445)	OR (95% CI)	
			Unadjusted	Adjusted ^b^
Achieved positive effect	119 (37.3)	319	1	1
Not achieved positive effect	56 (44.4)	126	1.35 (0.89–2.04)	1.32 (0.85–2.04)
*p* ^c^	0.172			

^a^ Abbreviations: GLS—global life satisfaction; OR—odds ratio; CI—confidence interval. ^b^ Adjusted for gender, age and family affluence. ^c^ *z* test.

## Abbreviations

The following abbreviations are used in this manuscript:

CI	Confidence Interval
CPQ	Child Perceptions Questionnaire
EWB	Emotional Well-being
FAS	Family Affluence Scale
FL	Functional Limitations
GLS	Global Life Satisfaction
HBSC	Health Behaviour in School-aged Children study
IQR	Interquartile Range
OHRQoL	Oral Health Related Quality of Life
OR	Odds Ratio
OS	Oral Symptoms
QoL	Quality of Life
SD	Standard Deviation
SWB	Social Well-being
WHO	World Health Organization
